# Experimental hut and bioassay evaluation of the residual activity of a polymer-enhanced suspension concentrate (SC-PE) formulation of deltamethrin for IRS use in the control of *Anopheles arabiensis*

**DOI:** 10.1186/s13071-014-0454-1

**Published:** 2014-10-02

**Authors:** Richard M Oxborough, Jovin Kitau, Rebecca Jones, Franklin W Mosha, Mark W Rowland

**Affiliations:** Department of Disease Control, London School of Hygiene and Tropical Medicine (LSHTM), London, UK; Department of Entomology and Parasitology, Kilimanjaro Christian Medical University College (KCMUCo) of Tumaini University, Moshi, Kilimanjaro Tanzania; Department of Entomology, Pan-African Malaria Vector Research Consortium, (PAMVERC), Moshi, Kilimanjaro Tanzania

**Keywords:** IRS, Deltamethrin, Pyrethroid, Long-lasting, *Anopheles arabiensis*, Vector control, Malaria, Experimental hut

## Abstract

**Background:**

The Stockholm Convention on Persistent Organic Pollutants (POPs) came into effect in 2004; the use of DDT for malaria control has been allowed to continue under exemption since then due to a perceived absence of equally effective and efficient alternatives. Alternative classes of insecticide for indoor residual spraying (IRS) have a relatively short residual duration of action (2-6 months according to WHO). In areas of year-round transmission multiple spray cycles are required, resulting in significantly higher costs for malaria control programs and user fatigue. This study evaluated performance of a new formulation of deltamethrin (pyrethroid) with polymer (SC-PE) to prolong the effective residual action to >6 months.

**Methods:**

Deltamethrin SC-PE was evaluated alongside an existing water dispersible granule (WG) formulation and DDT water dispersible powder (WP) in laboratory and hut bioassays on mud, concrete, palm thatch and plywood substrates. An experimental hut trial was conducted in Lower Moshi Rice Irrigation Zone, Tanzania from 2008-2009 against wild, free-flying, pyrethroid susceptible *An. arabiensis*. Performance was measured in terms of insecticide-induced mortality, and blood-feeding inhibition. Bioassays were carried out monthly on sprayed substrates to assess residual activity.

**Results:**

Bioassays in simple huts (designed for bioassay testing only) and experimental huts (designed for testing free flying mosquitoes) showed evidence that SC-PE improved longevity on mud and concrete over the WG formulation. Both deltamethrin SC-PE and WG outperformed DDT in bioassays on all substrates tested in the laboratory and simple huts. In experimental hut trials SC-PE, WG and DDT produced high levels of *An. arabiensis* mortality and the treatments were equivalent over nine months’ duration. Marked seasonal changes in mortality were recorded for DDT and deltamethrin treatments, and may have been partly influenced by outdoor temperature affecting indoor resting duration of mosquitoes on sprayed surfaces, although no clear correlation was demonstrated.

**Conclusions:**

There is a limited range of alternative insecticides for IRS, and deltamethrin SC-PE is likely to have an important role as part of a rotation strategy with one or more different insecticide classes rotated annually, particularly in areas that currently have low levels of pyrethroid resistance or low LLIN coverage and year-round malaria transmission.

## Background

IRS for malaria vector control has proven successful in substantially reducing transmission in a range of settings, both historically during the malaria eradication era of the 1950’s and 60’s, and more recently in meso- and holo-endemic countries in Africa [1-3]. Interruption of malaria transmission in the USA, partly through DDT house-spraying, led to the initiation of the Global Malaria Eradication Program in 1955 [4]. Enthusiasm that IRS with DDT could result in global malaria eradication led to the initiation of large-scale IRS programs in several countries. Between 1955-1978 malaria was eliminated from 37 countries, mostly in Europe and the Americas at the limits of global malaria transmission [4,5].

IRS was not taken to scale in most sub-Saharan malaria endemic countries during the global eradication campaign [6,7]. Southern Africa was the exception. IRS programs using DDT began in the 1960’s and were supported for several decades, with later introduction of pyrethroids and carbamates. Countries with sustained IRS activities in Africa, including South Africa, Zambia, Namibia, Swaziland, Zimbabwe and Botswana, achieved sizeable reductions in malaria vector populations and malaria incidence [7]. Focal IRS in the southern Africa region has remained important in areas of high malaria burden and areas at risk of epidemics. In 2007, about 14 million people in southern Africa were protected by IRS [6,7].

WHO has since reaffirmed the importance of IRS as a primary intervention for reducing or interrupting malaria transmission [8]. Funding for IRS in Africa has increased dramatically in recent years. The President’s Malaria Initiative (PMI) was launched in 2005 as a 5-year, $1.2 billion initiative to rapidly scale-up malaria prevention in 15 high-burden countries [9]. Mainly as a result of increased IRS funding from PMI, 8% (58 million people) of sub-Saharan Africa were protected by IRS in 2012 [10]. Notable recent examples of successful malaria control using pyrethroid IRS in Africa are São Tomé and Príncipe, and Zanzibar where IRS contributed to reducing malaria prevalence to less than 1% within 2 years of the 1^st^ application [11,12]. Pyrethroid resistance has spread rapidly in the past decade throughout sub-Saharan Africa, and many spray programmes have switched to the use of non-pyrethroid insecticides, mainly bendiocarb and pirimiphos-methyl [13]. However, the point at which pyrethroid resistance results in control failure has yet to be demonstrated and pyrethroids may still have an important role as part of a resistance management strategy involving rotation of IRS insecticides [14].

IRS has remained the dominant vector control strategy for malaria control in India since adoption of the strategy in 1953 [10]. In 2010, IRS with diethyldiphenyltrichloroethane (DDT), malathion and pyrethroids protected 53 million people, compared with only 9.5 million protected by ITNs [15]. Global use of vector control insecticides was dominated by DDT in terms of quantity applied (71% of total) and pyrethroids in terms of surface area covered (81% of total) between 2000-2009 [16]. The majority of DDT was sprayed in India, with usage remaining fairly constant between 2000-2009. Globally an average of 4,429 tonnes per year of DDT was used for residual spraying vector control during this time [16]. Of the insecticides recommended by the World Health Organization Pesticide Evaluation Scheme (WHOPES) for IRS, the longest-lasting is currently DDT, with duration of effective action greater than 6 months (according to WHO) [17]. The Stockholm Convention on persistent organic pollutants (2001) stipulates that, ‘countries using DDT are encouraged to reduce and eliminate the use of DDT over time and switch to alternative insecticides’ [18]. Despite this agreement, which became international law in 2004, global use of DDT has not changed substantially [16]. The use of DDT for malaria control has been allowed to continue under exemption since then and there is likely to be a continued role for DDT in malaria control until equally cost-effective alternatives are developed [19].

Bendiocarb is a commonly used alternative to DDT and pyrethroids, but can have a relatively short residual action of 2-6 months (according to WHOPES) and costs roughly 3 times more than pyrethroids (per 100m^2^ sprayed), [17,20,21]. In areas where the transmission season is >6 months, multiple spray rounds can become expensive, logistically demanding, and inconvenient to householders [8]. The residual lifespan of IRS insecticides is of key importance. LLINs have proved to be much more cost-effective than IRS programs with the average IRS cost per person/yr protected of $2.62 compared with $1.39 for 3-year duration LLINs [20]. Longer-lasting pyrethroid IRS could reduce the cost/person protected, which could in turn reduce reliance upon DDT in India.

Despite added impetus for the development of new public health insecticides, notably from the Innovative Vector Control Consortium (IVCC), alternative classes of insecticide for public health use are emerging slowly [22]. For continued cost-effectiveness of IRS programs it is important to develop new longer-lasting formulations of currently available insecticides [23]. There are several formulation options for pesticides designed to maximize biological efficacy and reduce harmful effects [24]. Encapsulation technology has been used to extend the residual performance of current WHO recommended IRS insecticides through slow release of core active ingredients, such as lambdacyhalothrin CS [17]. A recent successful example was a new CS formulation of the organophosphate, pirimphos-methyl, which extended residual duration from 2-3 months (for the EC formulation of the same active ingredient) to 4-6 months (according to WHO), [25,26]. Polymers have also been used to extend residual performance of public health pesticides, notably for textile treatments such as the “dip-it-yourself” deltamethrin mosquito net treatment K-O Tab® 1-2-3 [27].

Deltamethrin wettable powder (WP) and water dispersible granules (WG) have previously been recommended by WHOPES for IRS at a dosage range of 20-25mg/m^2^, with 3–6 months of expected duration of effective action [28]. In this study a new formulation of deltamethrin with SC-PE polymer was assessed for residual performance, with the aim being to exceed performance of the WG formulation and equal that of DDT [27].

## Methods

### Insecticide formulations

A new formulation of deltamethrin polymer-enhanced suspension concentrate (SC-PE) containing 62.5 g of active ingredient per litre (K-Othrin Polyzone®, Bayer CropScience, Monheim am Rhein, Germany) was evaluated alongside the existing deltamethrin water dispersible granule (WG) 250 g/kg (K-Othrin®, Bayer CropScience, Monheim am Rhein, Germany) and DDT wettable powder (WP) 750 g/kg (Avima, Johannesburg, South Africa).

### Laboratory assessment of residual performance

Cone bioassays, based on WHO guidelines, were conducted monthly on sprayed substrates of concrete, mud, and plywood to assess insecticidal duration of deltamethrin SC-PE, WG, and DDT WP [29]. Concrete was made using a ratio of 1:2 cement:sand and left to cure for a minimum of 4 weeks. Mud was made with a ratio of 2:3 soil:sand, using soil from Lower Moshi Field Station. Petri-dish size samples of concrete, mud and plywood substrates were sprayed with insecticide at an application rate of 40 ml/m^2^ [30] using a Potter Tower Precision Sprayer (Burkard Scientific, Uxbridge, UK) [29]. For each formulation three blocks were sprayed. Substrates were stored at ambient temperature and humidity (~20-28°C, 40-80% RH). Approximately 9 replicates of ~10 female *An. arabiensis* dondotha were tested each month with an exposure time of 30 minutes. After exposure, mosquitoes were transferred to 150 ml paper cups with 10% glucose solution provided *ad libitum*. Percentage mortality was scored after 24 h. *An. arabiensis* dondotha adult mosquitoes were insectary reared under controlled conditions of 22-27°C and 60-85% relative humidity. They were fully susceptible to deltamethrin when tested in WHO cylinder tests (100% mortality, deltamethrin 0.05%, n = 100).

### Field assessment of residual performance in simple huts

Simple huts were built corresponding to the design of experimental huts, minus the verandas [31]. The walls were lined with four types of material, with one material per wall surface: mud, concrete, plywood, palm thatch. There was an eave space, small windows and wooden ceiling to allow for ventilation and prevent extreme temperatures. Each spray treatment was tested using cone bioassays of insectary reared *An. arabiensis* 3-7 days after spraying and subsequently every month. Cones were randomly positioned every month and testing was done in the morning (06:30 – 10:00) when testing conditions were most suitable (i.e. humidity >60% RH, temperature <28°C). Mosquitoes were transferred to paper cups with access to 10% glucose solution and kept in the field station holding room with mortality recorded 24 h after testing.

The following treatments were sprayed in vertical swaths 71 cm wide marked with chalk on simple hut walls plastered with mud, concrete, palm thatch and plywood.

- Deltamethrin SC-PE, 50 mg ai/m^2^, (subsequently abbreviated to delta SC-PE 50)
- Deltamethrin SC-PE, 25 mg ai/m^2^, (subsequently abbreviated to delta SC-PE 25)
- Deltamethrin WG, 25 mg ai/m^2^, (subsequently abbreviated to delta WG 25)
- DDT WP, 2000 mg ai/m^2^, (subsequently abbreviated to DDT WP)
- Unsprayed

The walls were sprayed following the same protocol as the experimental huts. The duration of the vertical spray motion from ceiling to floor to achieve the required application rate was timed precisely and much practised by the spray person before he delivered the swath with the formulation at the requisite concentration.

### Indoor residual spraying experimental hut trials

Experimental hut trials were conducted at Kilimanjaro Christian Medical University College (KCMUCo) Harusini Field Station in Lower Moshi Rice Irrigation Zone (3°24′S, 37°21′E) where wild *An. arabiensis* and *Cx. quinquefasciatus* were the predominant man-biting mosquito species [32]. *An. arabiensis* densities were heavily dependent on rice cropping cycles. Wild *An. arabiensis* were tested in WHO cylinder tests with diagnostic dosages of permethrin, deltamethrin, lambdacyhalothrin and DDT papers (Vector Control Research Unit, Universiti Sains Malaysia) in April 2009, and a low frequency of resistance was detected (Table 1).

**Table 1:** **% mortality of wild collected semi-gravid** ***An. arabiensis*** **collected from surrounding cattle sheds**

**Insecticide**	**Concentration %**	**Number tested**	**Mortality %**
Deltamethrin	0.05	275	90
Permethrin	0.75	111	84
Lambdacyhalothrin	0.05	77	97
DDT	4	465	99

Experimental huts were constructed to a design described by the World Health Organization [29] and based on the original veranda hut design constructed in northern Tanzania [33,34]. Improvements were made involving a) reduction of eave gap to 5 cm, b) addition of inner ceiling board, c) concrete floor surrounded by a water filled moat [35]. The working principle of these huts has been described previously [31]. The experimental huts had either mud or concrete walls prepared to the specifications of laboratory blocks and simple hut walls. A palm thatched mat, typical of organic fibres used in some rural housing [36], was affixed to the ceiling before spraying. The walls and ceiling were sprayed with a Hudson sprayer (H.D. Hudson Manufacturing Company, Chicago, Illinois, USA) at an application rate of 40 ml/m^2^ [30]. A guidance pole was used to ensure a consistent vertical swath 71 cm wide and swath boundaries were marked out with chalk on walls and ceiling to improve spray accuracy. Verandas were protected during spraying by blocking the open eaves and windows with a double layer of plastic and Hessian sackcloth. A limitation was that no high performance liquid chromatography (HPLC) was conducted to confirm the dosages sprayed. However, the amount of insecticide remaining in the spray tank after spraying each hut indicated that application rates were within 20% of the target.

Ethical approval was granted from the review boards of LSHTM and Tanzania National Institute of Medical Research (NIMR/HQ/R.8c/Vol.I/24). Adult volunteers of 18 years or older were selected as volunteers from the local village to sleep in the huts overnight. The risks of malaria were explained and volunteers were provided with chemoprophylaxis, but taking was not enforced or observed. During the trial each volunteer was monitored daily for fever or possible adverse effects due to the IRS. Written informed consent was obtained from all volunteer sleepers and documented. Volunteers were given basic remuneration for participating in the study. It was explained they had the right to withdraw from the trial at any time without penalty. Adult volunteers slept in each hut nightly from 20:30-6:30. Sleepers were rotated between huts on successive nights to reduce any bias due to differences in individual attractiveness to mosquitoes. Mosquito collections were carried out using mouth aspirators between 6:30-08:00 each morning by trained field assistants. White sheets were laid on the concrete floor to make dead mosquitoes more easily visible. Dead mosquitoes were collected from the floor of verandas, window traps and bedroom. Live mosquitoes in the sprayed room were not collected in order to allow for natural resting times on treated surfaces, and were only collected after exiting to verandas or window traps. Live mosquitoes were transferred to 150 ml paper cups and provided with 10% glucose solution for scoring gonotrophic status and delayed mortality after 24h. All members of the *An. gambiae* species complex identified by morphological characteristics were assumed to be *An. arabiensis* based on PCR identification between 2005-2013, which showed the absence of *An. gambiae s.s.* [37-40].

The following treatments were sprayed in a total of 7 experimental huts.

- Deltamethrin SC-PE, 25 mg/m^2^ (one mud and one concrete walled hut)
- Deltamethrin WG, 25 mg/m^2^ (one mud and one concrete walled hut)
- DDT WP, 2000 mg/m^2^ (one mud and one concrete walled hut)
- Unsprayed (one mud walled hut)

### Analysis of residual performance in the laboratory

Treatments were compared according to the time interval since spray application for mortality to fall to 80% (based on WHOPES criteria) and 50% [29]. Mixed effect logistic regression models were used to fit mortality trajectories over time separately for each treatment (delta SC-PE 25 mg/m^2^, delta SC-PE 50 mg/m^2^, delta WG 25 mg/m^2^, and DDT WP 2000 mg/m^2^) and substrate (concrete and mud). All statistical modelling was performed on the log odds scale at the individual mosquito level and results back transformed to the proportion scale. There was little evidence of a departure from a linear decrease in the log odds of death over time so a linear term in time was specified as the only predictor in all models. A random effect was specified in all models to account for similarities in mosquitoes tested at the same time point and for potential behavioural clustering within the same test batch. The equations given by the estimates from the logistic regression models were solved to obtain estimates of the time points at which mortality fell to 80 and 50%. Ninety-five per cent confidence intervals (CI) were estimated using the bias corrected bootstrap method with 2,000 replications. Differences between treatments in estimated time for mortality to fall to 80 and 50% were calculated and statistically significant differences inferred from the bootstrap 95% CI (p = 0.05).

### Analysis of simple hut and experimental hut bioassays

Analysis of hut bioassays was similar to that described for laboratory bioassays. For wall assays, separate models were fitted for each hut. For ceiling assays, data from huts treated with the same insecticide (but with different wall materials) were combined.

### Analysis of experimental hut trial

The number of mosquitoes collected from the two closed verandas was multiplied by two to adjust for the unrecorded escapes through the two open verandas which were left unscreened to allow routes for entry of wild mosquitoes via the gaps under the eaves [9,24]. The data were analysed to show the effect of each treatment in terms of:

Overall mortality = Total proportion of mosquitoes dead on the morning of collection, plus delayed mortality after holding for a total of 24 hours.

Blood feeding inhibition = Percentage of blood-fed mosquitoes from a treated hut relative to percentage from the unsprayed negative control.

Mixed effect logistic regression models were used to fit mortality trajectories over time. All statistical modelling was performed on the log odds scale. The main predictors were hut treatment (each of delta SC-PE 25 mg/m^2^, delta WG 25 mg/m^2^ and DDT WP 2000 mg/m^2^ on both mud and concrete), polynomial terms in time, and interactions between treatment and each of the time terms. Modelling was done for the supplementary explanatory experimental hut studies with the added predictor of covering and uncovering the palm thatch ceiling. Mean indoor and outdoor overnight temperature and humidity were added as covariates in order to examine possible associations between mortality and climate factors. All models were adjusted for sleeper and included a random effect to account for similarities among mosquitoes entering huts on the same day and potential behavioural clustering.

## Results

Laboratory (mud, concrete), simple hut (mud, concrete), and experimental hut (mud, concrete, palm thatch) bioassay results indicating the duration of residual activity of the deltamethrin and DDT formulations are presented in Table 2. The differences in longevity are shown in Table 3, showing residual time (RT) taken for mortality to drop below 80% (RT 80) and 50% (RT 50).

**Table 2:** **Time for mortality to drop below 80% and 50% for laboratory, simple hut, and experimental hut bioassays**

**Substrate**	**Insecticide**	**Estimated time to 80% mortality**		**Estimated time to 50% mortality**	
		**Time (months)**	**95% CI**	**Time (months)**	**95% CI**
**Laboratory bioassays**					
**Mud**	Delta SC-PE 50	13.4	(12.8 to 14.3)	15.8	(15.0 to 17.1)
	Delta SC-PE 25	8.3	(7.5 to 9.1)	11.6	(10.9 to 12.4)
	Delta WG 25	8.1	(7.6 to 8.7)	10.9	(10.4 to 11.4)
	DDT WP 2000	5.2	(4.4 to 5.9)	8.4	(7.8 to 9.0)
**Concrete**	Delta SC-PE 50	†	†	†	†
	Delta SC-PE 25	15.5	(14.5 to 17.3)	†	†
	Delta WG 25	14.9	(13.8 to 16.9)	†	†
	DDT WP 2000	10.1	(8.9 to 11.4)	14.6	(13.3 to 16.6)
**Simple hut bioassays**					
**Mud**	Delta SC-PE 50	†	†	4.6	(2.4 to 6.0)
	Delta SC-PE 25	†	†	6.0	(5.0 to 6.9)
	Delta WG 25	†	†	2.6	(0.3 to 4.1)
**Concrete**	Delta SC-PE 50	11.2	(10.4 to 12.1)	14.7	(13.7 to 16.0)
	Delta SC-PE 25	8.0	(6.7 to 9.0)	12.4	(11.3 to 13.9)
	Delta WG 25	†	†	2.1	(†to 3.6)
**Experimental hut bioassays**					
**Mud**	Delta SC-PE 25	2.8	(0.2 to 4.6)	8.0	(6.7 to 9.2)
	Delta WG 25	†	†	0.5	(†to 3.0)
	DDT WP 2000	†	†	3.3	(1.1 to 5.0)
**Concrete**	Delta SC-PE 25	11.4	(9.2 to 16.7)	†	†
	Delta WG 25	5.8	(0.8 to 8.2)	†	†
	DDT WP 2000	7.0	(4.3 to 8.9)	12.0	(10.4 to 15.1)

***Notes*** *: † indicates that statistical models produced estimates outside the study period.*

**Table 3:** **Comparison of treatments for mortality to drop below 80% and 50% for laboratory, simple hut, and experimental hut bioassays**

**Substrate**	**Treatment comparison**	**Difference in estimated time to 80% mortality**			**Difference in estimated time to 50% mortality**		
		**Time (months)**	**95% CI**	**P-value**	**Time (months)**	**95% CI**	**P-value**
**Laboratory Bioassays**							
**Mud**	SC-PE 50 vs SC-PE 25	5.0	(4.0 to 6.2)	<0.05	4.2	(3.0 to 5.6)	<0.05
	SC-PE 50 vs WG	5.3	(4.4 to 6.3)	<0.05	4.9	(4.0 to 6.2)	<0.05
	SC-PE 50 vs DDT	8.2	(7.2 to 9.4)	<0.05	7.4	(6.4 to 8.7)	<0.05
	SC-PE 25 vs WG	0.2	(-0.8 to 1.2)	n/s	0.7	(-0.1 to 1.6)	n/s
	SC-PE 25 vs DDT	3.2	(2.1 to 4.3)	<0.05	3.2	(2.3 to 4.3)	<0.05
	WG vs DDT	2.9	(2.0 to 3.9)	<0.05	2.5	(1.7 to 3.2)	<0.05
**Concrete**	SC-PE 25 vs WG	0.6	(-1.5 to 2.5)	n/s	†	†	†
	SC-PE 25 vs DDT	5.4	(3.8 to 7.3)	<0.05	†	†	†
	WG vs DDT	4.8	(3.0 to 6.8)	<0.05	†	†	†
**Simple Hut Bioassays**							
**Mud**	SC-PE 50 vs SC-PE 25	†	†	†	−1.4	(0.4 to -3.7)	n/s
	SC-PE 50 vs WG	†	†	†	2.0	(-0.5 to 4.5)	n/s
	SC-PE 25 vs WG	†	†	†	3.4	(1.6 to 5.9)	<0.05
**Concrete**	SC-PE 50 vs SC-PE 25	3.2	(1.8 to 4.7)	<0.05	2.3	(0.5 to 4.0)	<0.05
	SC-PE 50 vs WG	†	†	†	12.6	(10.6 to 15.1)	<0.05
	SC-PE 25 vs WG	†	†	†	10.3	(8.3 to 13.0)	<0.05
**Experimental Hut Bioassays**							
**Mud**	SC-PE 25 vs WG	†	†	†	7.5	(4.4 to 13.8)	<0.05
	SC-PE 25 vs DDT	†	†	†	4.7	(2.6 to 7.2)	<0.05
	WG vs DDT	†	†	†	−2.8	(-9.9 to 0.5)	n/s
**Concrete**	SC-PE 25 vs WG	5.7	(1.9 to 11.6)	<0.05	†	†	†
	SC-PE 25 vs DDT	4.4	(1.3 to 9.5)	<0.05	†	†	†
	WG vs DDT	−1.2	(-5.9 to 2.4)	n/s	†	†	†

***Notes*** *: † indicates that statistical models produced estimates outside the study period.*

### Laboratory assessment of residual performance

**Figure 1:**
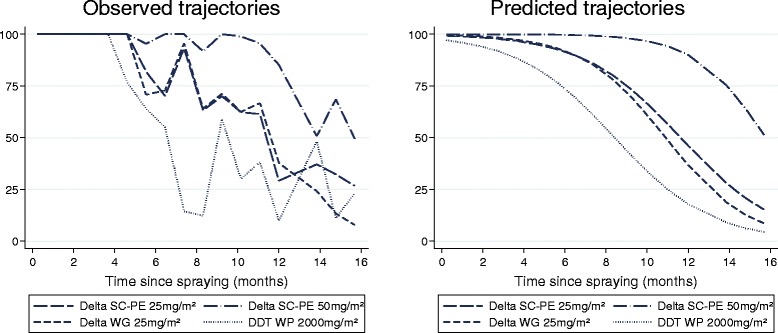
**% Mortality of** ***An. arabiensis*** **after 30 mins exposure in the laboratory to insecticide-treated mud blocks tested over 16 months.**

On mud, delta SC-PE 25 mg/m^2^ killed >80% of *An. arabiensis* for 8.3 months (95% CI: 7.5-9.1), but performed no better than the WG formulation (p > 0.05). Both SC-PE and WG formulations provided greater residual performance than DDT, which killed >80% for only 5.2 months (95% CI: 4.4-5.9). Delta SC-PE 50 mg/m^2^ lasted significantly longer than the SC-PE 25 and WG 25 treatments, with >80% mortality achieved for 13.4 months, (12.8-14.3) (p < 0.05) (Figure 1).

**Figure 2:**
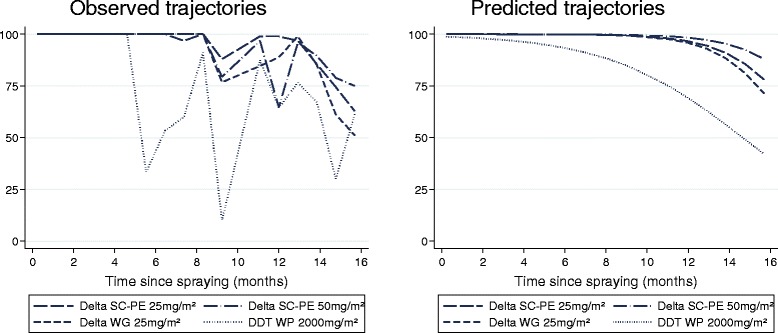
**% Mortality of** ***An. arabiensis*** **after 30 mins exposure in the laboratory to insecticide-treated concrete blocks tested over 16 months.**

On concrete, delta SC-PE 25 killed >80% of *An. arabiensis* for 15.5 months (95% CI: 14.5-17.3), but performed no better than the WG formulation (p > 0.05). Both the SC-PE 25 and WG 25 lasted longer than DDT (p < 0.05), which killed >80% for only 10.1 months (95% CI: 8.9-11.4). Statistical comparison with SC-PE 50 could not be made as mortality remained above 80% for the duration of the study (Figure 2). On plywood, all formulations killed >95% of *An. arabiensis* 16 months after spraying (data not presented).

### Field assessment of residual performance in simple huts

RT80 is not presented for formulations sprayed on mud as mortality was already below 80% when bioassays were conducted < 1 week after spraying (Table 2). Delta SC-PE 25 killed >50% of *An. arabiensis* for 6.0 months (95% CI: 5.0-6.9) and lasted significantly longer than the WG (p < 0.05) but was no different to the SC-PE 50 (p > 0.05). Mortality for DDT was <50% <1 week after spraying and was not included in the analysis.

On concrete, delta SC-PE 25 killed >80% of *An. arabiensis* for 8.0 months (95% CI: 6.7-9.0) and >50% for 12.4 months (95% CI: 11.3-13.9) and lasted significantly longer than the WG which only killed >50% for 2.1 months (p < 0.05) (Table 3). The SC-PE 50 lasted longer than both SC-PE 25 and WG 25 (p < 0.05). Mortality for DDT was surprisingly low and neither RT 80 nor 50 could be estimated.

Bioassays done on plywood and palm thatch produced very high levels of mortality for all deltamethrin formulations, with little loss of activity over the duration of the trial; therefore analysis of RT 80 and RT 50 was not done. On plywood, observed mortality was >80% for SC-PE 25 and WG 25 for 12 months and 18 months for SC-PE 50. On palm thatch observed mortality for SC-PE 25 and WG 25 was >80% for 14 months, compared with 18 months for SC-PE 50, while DDT produced surprisingly low levels of observed mortality with >80% for only 2 months.

### Residual activity of formulations in experimental huts

**Figure 3:**
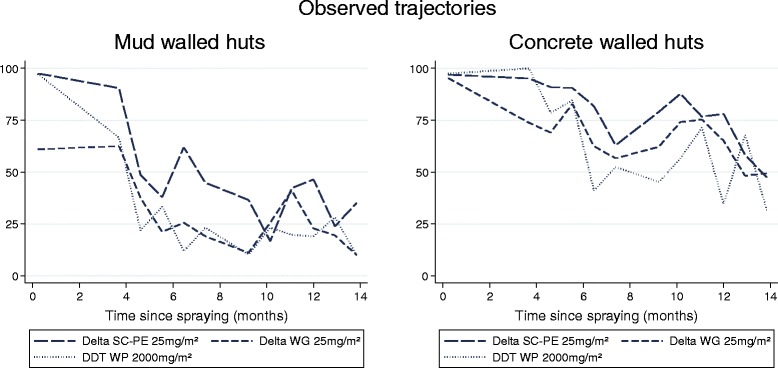
**WHO cone bioassays on experimental hut walls showing %** ***An. arabiensis*** **mortality tested up to 14 months after spraying (observed results).**

**Figure 4:**
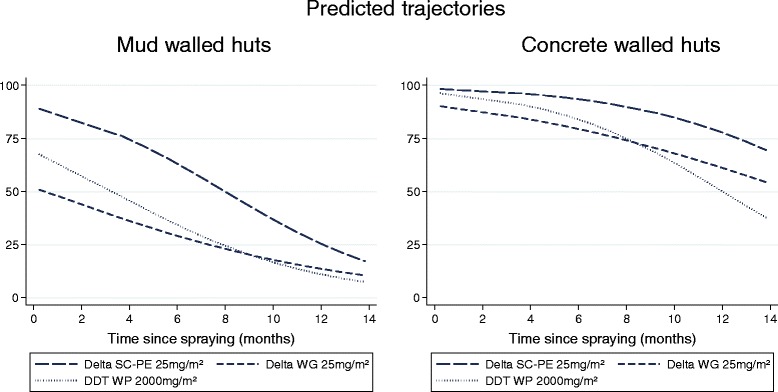
**WHO cone bioassays on experimental hut walls showing %** ***An. arabiensis*** **mortality tested up to 14 months after spraying (predicted results).**

WHO cone bioassays on walls of experimental huts showed consistently higher mortality for all formulations on concrete than on mud. On mud, only RT 50 was compared as mortality dropped below 80% shortly after spraying. The SC-PE 25 killed >50% of *An. arabiensis* for 8.0 months (95% CI: 6.7-9.2) and showed greater longevity than WG which produced an RT50 of only 0.5 months (95% CI: †-3.0) and DDT (p < 0.05) (Table 3, Figures 3, and 4).

On concrete, the SC-PE 25 formulation was the longest lasting and killed >80% of *An. arabiensis* for 11.4 months (95% CI: 9.2-16.7) compared with 5.8 months for WG (95% CI: 0.8-8.2) and 7.0 months for DDT (95% CI: 4.3-8.9) (p < 0.05) (Table 2, and 3; Figures 3, and 4).

**Figure 5:**
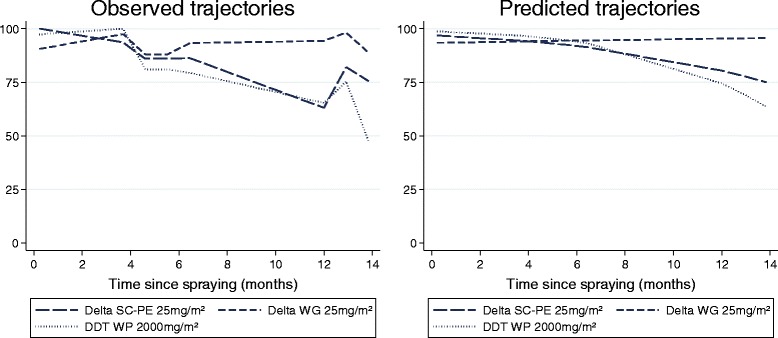
**WHO cone bioassays on experimental hut ceiling showing %** ***An. arabiensis*** **mortality tested 14 months after spray application.**

Observed and predicted mortality curves are presented in Figure 5 for bioassays on sprayed palm thatch ceiling in experimental huts. As in simple hut bioassays, mortality was stable and no loss of activity was recorded for the SC-PE 25, up to14 months after spraying (Figure 5). DDT and delta WG followed a similar trajectory but showed a slight decrease in mortality between 6 and 14 months, although mortality was still >60% after 14 months.

### Experimental hut trial against wild, free flying, *An. arabiensis* over 9 months to compare efficacy of DDT and deltamethrin formulations

**Figure 6:**
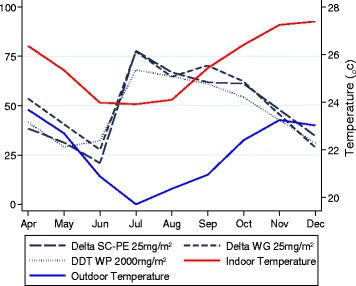
**Trend of mean monthly temperature at the experimental hut site in relation to percentage mortality with DDT, deltamethrin WG and SC-PE.** Notes: No data was collected for November. Data was combined for mud & concrete walled huts and presented by treatment.

Mortality of free-flying, wild *An. arabiensis* showed an unusual trend during the course of the trial and peaked 4 months after spraying (Figure 6). Mortality of wild *An. arabiensis* during the first month after spraying was relatively low for all treatments (40-55% across treatments). Mortality rates continued to fall over the next three months (April-June). Four months after spraying (July) mortality rates suddenly increased and reached a peak with 75% (95% CI: 70-80) (mud) and 80% (95% CI: 75-84) (concrete) mortality recorded for delta SC-PE 25 (Table 4). Between 5-9 months after spraying (August-December) there was a gradual decrease in mortality for all treatments with mortality <45% nine months after spraying. There was no evidence of any effect of treatment on mortality trajectories over time (P > 0.05) although there was weak evidence that average mortality levels were slightly higher in concrete than mud huts (p = 0.071). Rather more expectedly, cone bioassay results on hut walls showed highest mortality shortly after spraying and a trend of declining insecticidal activity over time (Figures 3, and 4).

**Table 4:** **Experimental hut summary results for wild free-flying** ***An. Arabiensis*** **during the 9 month efficacy trial**

**Insecticide (Wall) substrate)**	**Outcome measure**	**Time after spraying (months)**							
		**April**	**May**	**June**	**July**	**August**	**September**	**October**	**December**
**Delta SC-PE** 25 mg/m^2^ (Mud)	Number Collected	76	88	69	252	791	439	225	30
	**% Mortality**	**28**	**34**	**13**	**75**	**66**	**59**	**56**	**37**
	95% CI	19-39	25-45	7-23	70-80	62-69	54-63	49-62	22-55
	% Blood-fed	71	64	36	19	21	31	39	53
	**% Blood-feeding inhibition**	**19**	**35**	**60**	**68**	**54**	**58**	**45**	**45**
**Delta WG** 25 mg/m^2^ (Mud)	Number Collected	65	88	32	338	850	397	234	30
	**% Mortality**	**40**	**43**	**19**	**72**	**67**	**71**	**63**	**23**
	95% CI	29-52	33-54	9-36	67-77	64-70	66-75	56-69	12-42
	% Blood-fed	77	52	34	17	25	21	27	80
	**% Blood-feeding inhibition**	**13**	**47**	**62**	**71**	**46**	**71**	**62**	**17**
**DDT WP** 2000 mg/m^2^ (Mud)	Number Collected	20	48	102	348	850	444	174	23
	**% Mortality**	**40**	**29**	**30**	**66**	**70**	**60**	**59**	**44**
	95% CI	21-62	18-43	22-40	61-71	67-73	56-65	52-66	25-64
	% Blood-fed	60	42	37	20	29	33	33	61
	**% Blood-feeding inhibition**	**32**	**57**	**58**	**66**	**37**	**55**	**54**	**36**
**Delta SC-PE** 25 mg/m^2^ (Concrete)	Number Collected	83	94	103	343	937	476	200	57
	**% Mortality**	**48**	**29**	**26**	**80**	**68**	**65**	**67**	**28**
	95% CI	38-59	21-39	19-36	75-84	65-71	60-69	60-73	18-41
	% Blood-fed	75	67	53	20	22	31	36	39
	**% Blood-feeding inhibition**	**15**	**32**	**40**	**66**	**52**	**58**	**49**	**59**
**Delta WG** 25 mg/m^2^ (Concrete)	Number Collected	75	65	44	323	947	383	272	26
	**% Mortality**	**65**	**37**	**34**	**83**	**62**	**70**	**62**	**39**
	95% CI	54-75	26-49	22-49	79-87	59-65	65-74	56-67	22-58
	% Blood-fed	64	49	48	17	19	22	33	23
	**% Blood-feeding inhibition**	**27**	**50**	**46**	**71**	**59**	**70**	**54**	**76**
**DDT WP** 2000 mg/m^2^ (Concrete)	Number Collected	69	83	109	371	1105	454	233	26
	**% Mortality**	**42**	**29**	**34**	**70**	**61**	**62**	**51**	**27**
	95% CI	31-54	20-40	26-43	66-75	58-64	57-66	44-57	13-47
	% Blood-fed	59	61	47	18	21	28	34	54
	**% Blood-feeding inhibition**	**33**	**38**	**47**	**69**	**54**	**62**	**52**	**44**
**Untreated** (Mud)	Number Collected	50	57	47	161	255	111	86	23
	**% Mortality**	**16**	**4**	**6**	**17**	**11**	**2**	**1**	**4**
	95% CI	8-29	1-13	2-18	12-24	7-15	1-7	0-8	1-25
	% Blood-fed	88	98	89	59	46	73	71	96

**Figure 7:**
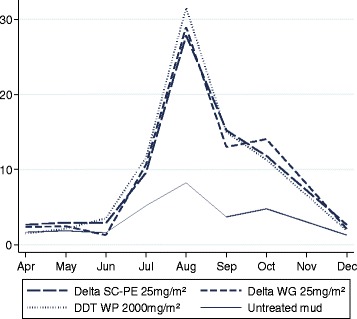
**Mean number of mosquitoes collected per night for experimental huts sprayed with DDT, deltamethrin WG and SC-PE.** Notes: No data was collected for November. Data was combined for mud & concrete walled huts and presented by treatment*.*

Climate data recorded at the field station (USB Wireless Touchscreen Weather Forecaster, Maplin, UK) showed that mean night temperature (from 20:30 to 6:30 h) was lowest during the cool season between June-September, 3-6 months after spraying, with indoor temperature ~24-25°C and outdoor ~20-21°C (Figure 6). After accounting for mortality trajectories over time, there was no evidence of any association between overnight temperature or humidity and mortality (P > 0.05). The number of *An. arabiensis* collected per day from huts was dependent on rice cropping cycles with peak numbers occurring between July and October (Figure 7).

Percentage blood-feeding was high in the unsprayed hut but varied by month between 46-98% (Table 4); the rate was lowest during August when mosquito densities were highest. All IRS treatments provided a considerable degree of personal protection, but the degree of protection varied over time. Peak blood-feeding inhibition was in July (four months after spraying) and ranged between 66-71% by treatment compared to the unsprayed control. Over the nine month trial 76-80% of *An. arabiensis* killed by the three treatments were unfed. The number of mosquitoes collected over the trial was substantially lower in the unsprayed control at 790 *An. arabiensis* females, compared with 1970 (mud) and 2293 (concrete) for delta SC-PE 25; 2034 (mud) and 2135 (concrete) delta WG 25; and 2009 (mud) and 2450 (concrete) for DDT. This probably indicates that a proportion of live mosquitoes were able to exit through open eaves. Insecticide-induced mortality in sprayed huts is likely to have limited the number of escapees. This should not affect the proportional comparisons between treatment, but may affect the overall mortality rates.

### Supplementary explanatory experimental hut testing

Bioassays in experimental huts (Figure 5) indicated high levels of mortality (>80%) for all formulations on palm thatch ceiling nine months after spraying, but much lower mortality for concrete and mud walls (Figures 3, and 4). Mortality achieved through mosquitoes contacting the palm thatch ceiling may have masked any differences in performance of wall substrates. Between 11-15 months after spraying a weekly rotation was done in all huts to cover/uncover the palm thatch ceiling with untreated plastic sheeting. Results are presented in Table 5. Surprisingly, covering the ceiling had no significant effect on % mortality for all formulations and substrates tested (P = 0.133-0.731). Between months 16-17 after spraying, the walls and ceiling of all mud-walled huts were covered with unsprayed plastic sheeting, while concrete-walled huts were left uncovered. This was done to investigate the possibility that mosquitoes may have been exiting other huts (with concrete walls) having picked up a lethal dosage of insecticide and dying in a nearby hut. Mortality was 3% for all three treated huts with covered walls and ceiling, 2% in the unsprayed control, but in uncovered concrete-walled huts mortality was 41%, 44%, and 42% respectively for delta SC-PE 25, WG 25, and DDT (Table 5). After 18 months the plastic sheeting was removed and mortality in the mud-walled huts returned to levels seen previously at 42%, 36%, and 36% respectively, indicating that mortality was caused by the treated surfaces in each individual hut and not as a result of mosquito movement.

**Table 5:** **Experimental hut summary results for wild free-flying** ***An. arabiensis during the supplementary experiments***

**Insecticide (wall substrate)**	**Outcome measure**	**Number of months after spraying**			
		**11-15 uncovered**	**11-15 ceiling covered**	**16-17 walls and ceiling covered †**	**18 uncovered**
**Delta SC-PE** 25 mg/m^2^ (Mud)	Number collected	365	499	521	183
	**% Mortality**	**41**	**37**	**3†**	**42**
	95% CI	31-52	28-48	1-6	35-50
	% Blood-fed	40	36	56	32
	**% Blood-feeding inhibition**	**42**	**33**	**5**	**20**
**Delta WG** 25 mg/m^2^ (Mud)	Number collected	300	559	463	130
	**% Mortality**	**46**	**33**	**3†**	**36**
	95% CI	31-61	24-43	1-7	28-45
	% Blood-fed	45	29	51	33
	**% Blood-feeding inhibition**	**35**	**46**	**14**	**18**
**DDT WP** 2000 mg/m^2^ (Mud)	Number collected	218	305	190	214
	**% Mortality**	**51**	**37**	**3†**	**36**
	95% CI	39-62	25-52	1-11	28-45
	% Blood-fed	35	37	80	38
	**% Blood-feeding inhibition**	**49**	**32**	**0**	**3**
**Delta SC-PE** 25 mg/m^2^ (Concrete)	Number collected	373	659	715	160
	**% Mortality**	**28**	**37**	**41**	**39**
	95% CI	22-34	28-48	34-48	30-49
	% Blood-fed	48	39	52	43
	**% Blood-feeding inhibition**	**30**	**28**	**12**	**0**
**Delta WG** 25 mg/m^2^ (Concrete)	Number collected	310	528	759	152
	**% Mortality**	**41**	**37**	**44**	**42**
	95% CI	27-57	30-44	37-52	33-52
	% Blood-fed	32	32	56	39
	**% Blood-feeding inhibition**	**54**	**41**	**5**	**3**
**DDT WP** 2000 mg/m^2^ (Concrete)	Number collected	262	508	705	174
	**% Mortality**	**49**	**44**	**42**	**40**
	95% CI	37-61	34-54	35-48	28-52
	% Blood-fed	44	34	58	33
	**% Blood-feeding inhibition**	**36**	**37**	**2**	**18**
**Untreated** (Mud)	Number collected	276	369	376	98
	**% Mortality**	**7**	**12**	**2†**	**2**
	95% CI	3-16	7-19	0-7	1-8
	% Blood-fed	69	54	59	40

***Notes*** *: †Indicates that the sprayed walls and ceiling of the experimental hut were covered with untreated plastic sheeting.*

## Discussion

The delta SC-PE 50 formulation was only tested in laboratory bioassays but showed improved longevity over delta SC-PE 25 and WG. This improved longevity over SC-PE 25 was most likely dosage related. The primary objective of this study was to determine whether delta SC-PE 25 formulation would achieve greater longevity than delta WG 25 and DDT WP when sprayed as IRS. Cone tests conducted on laboratory sprayed blocks showed that delta SC-PE 25 performed no better than the WG 25 formulation on mud, plywood and concrete substrates. In experimental hut and simple hut cone bioassays SC-PE 25 was significantly longer lasting than WG 25 on mud and concrete substrates but not on palm thatch or plywood.

Delta SC-PE 25 and WG 25 both lasted marginally longer than DDT in laboratory bioassays on mud and concrete and in simple hut bioassays on mud, concrete, palm thatch, and plywood.

In experimental hut cone tests over 14 months the delta SC-PE outperformed DDT on mud and concrete walls. Despite the majority of bioassay results indicating the SC-PE and WG outperformed DDT, there was no difference in performance against wild free-flying *An. arabiensis.* Delta SC-PE, WG 25 and DDT were equivalent and produced effective control of *An. arabiensis* for several months. Cone tests on hut walls indicated a gradual decline in mortality on concrete and a much more rapid decline on mud walls for delta SC-PE 25, WG 25 and DDT. The loss of activity on mud walls could have been masked by greater residual activity on the sprayed palm thatch ceiling, as thatch killed high proportions in cone tests 12 months after spraying. However, covering of the ceiling between months 11-15 with untreated plastic sheeting produced no difference in mortality, and indicated that the sprayed walls were still making a significant contribution to mortality. Further supplementary tests covering both the walls and ceiling of selected huts indicated that mortality was being caused by mosquitoes resting on walls and ceiling and ruled out the possibility of mosquitoes flying between huts before dying. Nevertheless, this raises an important issue surrounding substrates used in experimental hut IRS trials. Usually spraying is done on multiple substrates (walls, ceiling, and door) in the same experimental hut but the performance on a more favourable substrate (eg. palm thatch) may mask poor performance on another (eg. mud) [29]. Recent studies of house design indicated that ceilings are not common in some rural areas of Africa [41,42]. It was also observed during a recent IRS campaign near Lake Victoria, Tanzania that only the walls were routinely sprayed, while the roof beams were left unsprayed (when no ceiling was present) (Oxborough, personal observation). Therefore, it is critically important to determine the performance of new insecticides in experimental huts where only one substrate is sprayed and WHOPES guidelines may need updating accordingly.

The mortality trends for wild free-flying *An. arabiensis* were unexpected and appear to be influenced by factors other than insecticide sorption and degradation. Nevertheless, the overall trends were maintained within insecticide formulations throughout the trial. The reasons for seasonal fluctuations in mortality are most likely, in part, related to changes of temperature, although a clear correlation could not be shown. DDT and pyrethroid insecticides interfere with sodium and potassium conductance through nerve membranes and both show a negative temperature co-efficient with toxicity for the majority of insect species evaluated including *Anopheles* mosquitoes [43,44], cockroaches [45-47], tsetse flies [48], stored grain pests [49], and houseflies [50,51]. This appears to be due to greater nerve sensitivity as insecticide penetration is conversely greater at higher temperature [50].

Residual house spraying is only effective if the mosquito species concerned is endophilic and rests on the insecticide-treated surfaces for a sufficient time to pick up a lethal dose [52]. Changes in resting behaviour in response to seasonal changes in climate may have an important bearing on efficacy. *An. gambiae* gonotrophic cycle duration is closely correlated with temperature and it is likely that selecting a warmer microclimate while processing a blood-meal to eggs is advantageous in terms of natural selection [53]. At higher altitude where differences between indoor and outdoor temperature are greatest, indoor resting is more common [54-56]. It is conceivable that when outdoor temperature is low, IRS becomes more effective, due to mosquitoes spending relatively longer time resting on treated surfaces indoors. Resting behaviour appears to be relatively plastic, particularly for *An. arabiensis* [54], and may change according to season. As there was no straightforward statistical correlation between temperature and mortality, it is likely that several factors were involved, which could not be fully explained by this study. The initial high dosage of insecticide shortly after spraying may have partially overridden any temperature-related effects on mortality. Excito-repellent behaviour caused by DDT and deltamethrin is another factor, which will undoubtedly have had an impact on resting times on treated surfaces and time of exiting [57,58].

The months of highest percentage mortality coincided with the months of highest mosquito density when the rice fields were flooded and at their most productive. The high densities entering the huts in July-August would have been younger than at the tail end of the previous cropping season (April-June) when mortality was notably lower. There is an association between resistance to pyrethroids and age of adult mosquitoes, but the relationship is an inverse one, with mosquitoes tending to show reduced resistance as they get older. *An arabiensis* from Lower Moshi shows low grade metabolic resistance to permethrin and deltamethrin associated with increased expression of CYP4G16 oxidases and ABC2060 transporters [39,59] and studies on *An. gambiae* which carry CYP4G16 and other cytochrome P450s show greatest resistance when they are young [60]. The trends in this study are the opposite of what one might expect to see from a young population and so the explanation must lie elsewhere.

Most experimental hut studies of IRS insecticides have been done over a short duration of 2-3 months. The duration of this study has identified long-term factors, such as climate, which should be considered and investigated in more detail. This may have wider implications to national control programs that conduct IRS and highlights the need for proper monitoring of vector control interventions. In this study the low levels of mortality recorded between 1-3 months after spraying correlated with a time when mosquito numbers were relatively low, while peak mortality occurred when mosquito numbers were highest. If a temporary loss of control occurs for reasons other than insecticide decay, it is likely to be of minimal consequence so long as IRS is effective during peak mosquito and malaria transmission seasons.

According to WHOPES, DDT has the greatest longevity of all IRS recommended insecticides, with a duration of effective action of >6 months [17]. Delta WG is considered by WHOPES to be inferior to DDT with a residual action of 3-6 months. In this study both delta SC-PE and WG 25 formulations were equivalent or better than DDT in hut trials and cone bioassays. The Stockholm Convention on persistent organic pollutants came into effect in 2004 and stipulates that ‘countries using DDT are encouraged to reduce and eliminate the use of DDT over time and switch to alternative insecticides’ [18]. Despite this international agreement, global use of DDT has not changed substantially [16]. DDT is still used mainly due to longevity and low cost. The present study has shown that delta SC-PE or WG are comparable with DDT in terms of longevity. Delta WG is relatively inexpensive (and is not subject to the same additional costs for environmental management as DDT) and the overall cost of spray operations in Africa using deltamethrin or DDT have been shown to be comparable [61].

Pyrethroid use in Africa for IRS and LLIN has increased greatly between 2002- 2013 [16] and has probably accelerated the development and spread of pyrethroid resistance [62,63]. Of 17 African countries sprayed within the President’s Malaria Initiative (PMI)-funded IRS in 2012, only one was classified as having pyrethroid susceptible anophelines; the remainder had confirmed or emerging resistance [64]. WHO recommends that in areas of high LLIN coverage, pyrethroid insecticides should not be used for IRS as this will contribute to selection pressure [65]. This strategy has been adopted by some national control programmes, such as in Senegal, where pyrethroids are advocated for LLIN but not IRS, for better resistance management [66]. The long term strategy is to reduce reliance on the persistent organic pollutant (POP) DDT [18] and to reduce selection pressure on LLINs by reducing pyrethroid IRS use [65]. However, there is currently a shortage of alternative insecticides for IRS [22,23], and pyrethroid insecticides are likely to have an important role as part of a rotation strategy with one or more different insecticide classes rotated annually; particularly in areas that currently have low levels of pyrethroid resistance [65] or low LLIN coverage, such as India. The level of insecticide resistance at which effectiveness is compromised remains unknown and there is evidence to suggest that pyrethroids can reduce sporozoite rates by killing older mosquitoes, which become less resistant with age [60,67]. Deltamethrin SC-PE recently received recommendation by WHO for IRS at a dosage of 20-25 mg/m^2^, with an expected residual efficacy of 6 months [25].

## Conclusions

Deltamethrin IRS should be used judiciously as part of a resistance management strategy in rotation with other classes of IRS such as bendiocarb [68,69] and pirimiphos-methyl CS [26,70] according to GPIRM [14,65].
